# Anuric Acute Kidney Injury Due to Infection-related Glomerulonephritis Secondary to Foot Osteomyelitis

**DOI:** 10.7759/cureus.4476

**Published:** 2019-04-16

**Authors:** Alexander Lewis, Mitchell K Ng, Matthew Lacey, Amanda L Pensiero

**Affiliations:** 1 Neurology, University Hospitals Cleveland Medical Center, Cleveland, USA; 2 Internal Medicine, Case Western Reserve University School of Medicine, Cleveland, USA; 3 Internal Medicine, University Hospitals Cleveland Medical Center, Cleveland, USA

**Keywords:** infection-related glomerulonephritis, osteomyelitis, anuria, acute kidney injury

## Abstract

We report the case of a 76-year-old male who developed anuric acute kidney injury from infection-related glomerulonephritis (IRGN) secondary to foot osteomyelitis, confirmed via renal biopsy. The patient initially presented with wound dehiscence of the left foot following transmetatarsal amputation and Lisfranc operation in the setting of osteomyelitis. Hospitalization was complicated by worsening acute kidney injury requiring the initiation of hemodialysis. Despite successful surgical correction and the removal of the infectious source, the patient was unable to regain significant renal function and remained hemodialysis dependent. This case highlights a rarely seen complication of osteomyelitis and underscores the value of prompt and aggressive management of osteomyelitis in patients with infection-related glomerulonephritis.

## Introduction

Infection-related glomerulonephritis (IRGN) is defined as immune-mediated glomerular injury that arises in response to extra-renal bacterial infection [1]. IRGN, previously known as post-infectious glomerulonephritis, typically presents in children of one to four weeks following a streptococcal upper respiratory infection (the most well-known example being post-streptococcal glomerulonephritis (PSGN) or cutaneous skin infection [2-3]. However, over the past three decades, the epidemiology has shifted, and a significant proportion of cases currently occur in adults. For example, a recent review of 86 patients with renal-biopsy confirmed IRGN found in adults over 55, in 54.6% of cases [4]. In response, the term infection-related glomerulonephritis was coined, as adult cases of IRGN often occur concurrently, or precede, the diagnosis of extra-renal infection as compared to post-infection, as classically seen in children [1,3].

The estimated incidence of IRGN in adults is 0.3 and two cases per 100,000 person-years in developed and developing countries, respectively. This is in contrast to six and 24.3 cases per 100,000 person-years observed in children [5]. Known risk factors for IRGN include diabetes, alcoholism, malignancy, acquired immunodeficiency syndrome (AIDS), tuberculosis, and intravenous (IV) drug use. Primary sites of extrarenal infection responsible for adult IRGN are diverse and include upper respiratory tract, lung, heart, urinary tract, skin, teeth, and bone. In particular, osteomyelitis is not classically associated with glomerular disease and is believed to be responsible for <10% of IRGN, with a paucity of documented cases. We describe an episode of anuric acute kidney injury (AKI) with renal biopsy revealing diffuse endocapillary proliferative glomerulonephritis consistent with IRGN, occurring concurrently with foot osteomyelitis caused by methicillin-sensitive Staphylococcus aureus. Despite appropriate antibiotic therapy and successful surgical resection of the presumed source, the infection and the immune complexes thereafter had already seeded the kidney and the patient was unable to regain significant renal function.

## Case presentation

A 76-year-old Caucasian male with a history of chronic kidney disease (CKD) stage 3, type 2 diabetes complicated by neuropathy and retinopathy (HbA1c 8.6%), atrial fibrillation on dabigatran, hypertension (HTN), coronary artery disease status post (s/p) three-vessel coronary artery bypass grafting (CABG), heart failure with preserved ejection fraction (HFpEF), peripheral artery disease (PAD) with prior right below knee amputation (BKA) and recent left lower extremity transmetatarsal amputation, and Lisfranc amputation presented due to worsening left foot wound dehiscence. The patient was seen by podiatry prior to initial presentation, where left foot osteomyelitis was suspected given the worsening discharge, odor, and erythema. In consultation with vascular surgery, the left foot was deemed unsalvageable and amputation was recommended.

Initial vitals were significant for temperature 100.3 degrees Fahrenheit (normal range 97.7-99.5 degrees Fahrenheit), pulse 79 (normal range 60-100), blood pressure 138/70 (normal 120/80), respiratory rate 20 (normal range 12-20), with 94% oxygen saturation on room air (normal range 95-100%). On admission, labs were significant for a normal white blood cell (WBC) count of 8.6 K/cmm (normal range 3.6-11.0) with evidence of acute kidney injury (AKI) superimposed on chronic kidney disease (CKD) Stage 3 with a creatinine of 1.7 mg/dL (patient's baseline of 1.1 mg/dL, normal range 0.7-1.5 mg/dL). Radiographic studies of the affected foot revealed air pockets distal to the second and third cuneiforms, felt to represent the extension of deep wounds, raising concerns for chronic osteomyelitis. The patient was started on vancomycin, aztreonam, and metronidazole, given a prior history of piperacillin/tazobactam allergy, and was admitted to medicine for further management. During hospitalization, the patient’s kidney function continued to worsen (Cr: 1.7 mg/dL -> 2.7 -> 3.7 -> 4.4 -> 4.7 -> 5.7 mg/dL) with poor urine output (100-150 cubic centimeters (cc) daily or 0.05-0.07 mL/kg/hr, normal range 800-2000 mL or 1-2 mL/kg/hr). Blood cultures showed 48 hours no growth to date (NGTD) while wound cultures were positive for Staphylococcus aureus with gram-negative rods. Given worsening AKI and positive wound cultures, infectious disease and nephrology were consulted. With concern for medication-induced AKI, the decision was made to switch the patient to monotherapy with intravenous (IV) ertapenem 500 mg daily. However, the patient’s renal function continued to deteriorate, a temporary hemodialysis catheter was placed, and he was initiated on hemodialysis. The differential for the etiology for this patient’s AKI was extensive and included vancomycin-induced nephrotoxicity, obstructive causes (ruled out with no hydronephrosis seen on renal ultrasound and normal bladder scan), vascular causes (no renal artery stenosis or arteriolosclerotic occlusive disease observed on renal artery Doppler ultrasound), autoimmune causes (anti-nuclear antibody (ANA) negative), and vasculitides (antineutrophil cytoplasmic antibody (ANCA) studies negative). The patient required aggressive fluid removal with hemodialysis and ultrafiltration as he remained volume overload and continued to require supplemental oxygen.

**Figure 1:**
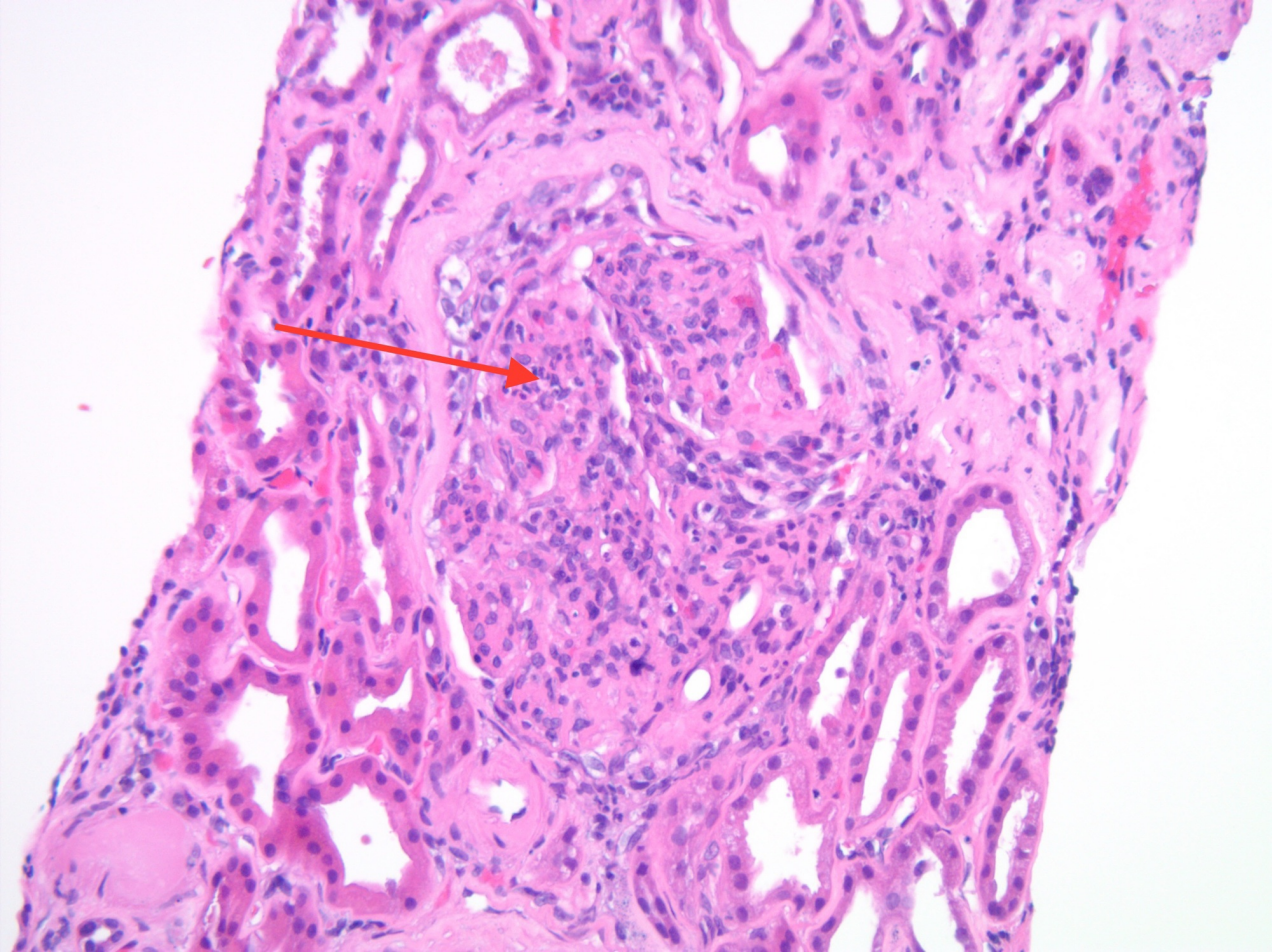
Light microscopy of renal biopsy demonstrating diffuse endocapillary proliferative and exudative glomerulonephritis and mesangial cell proliferation (as indicated by the red arrow)

**Figure 2:**
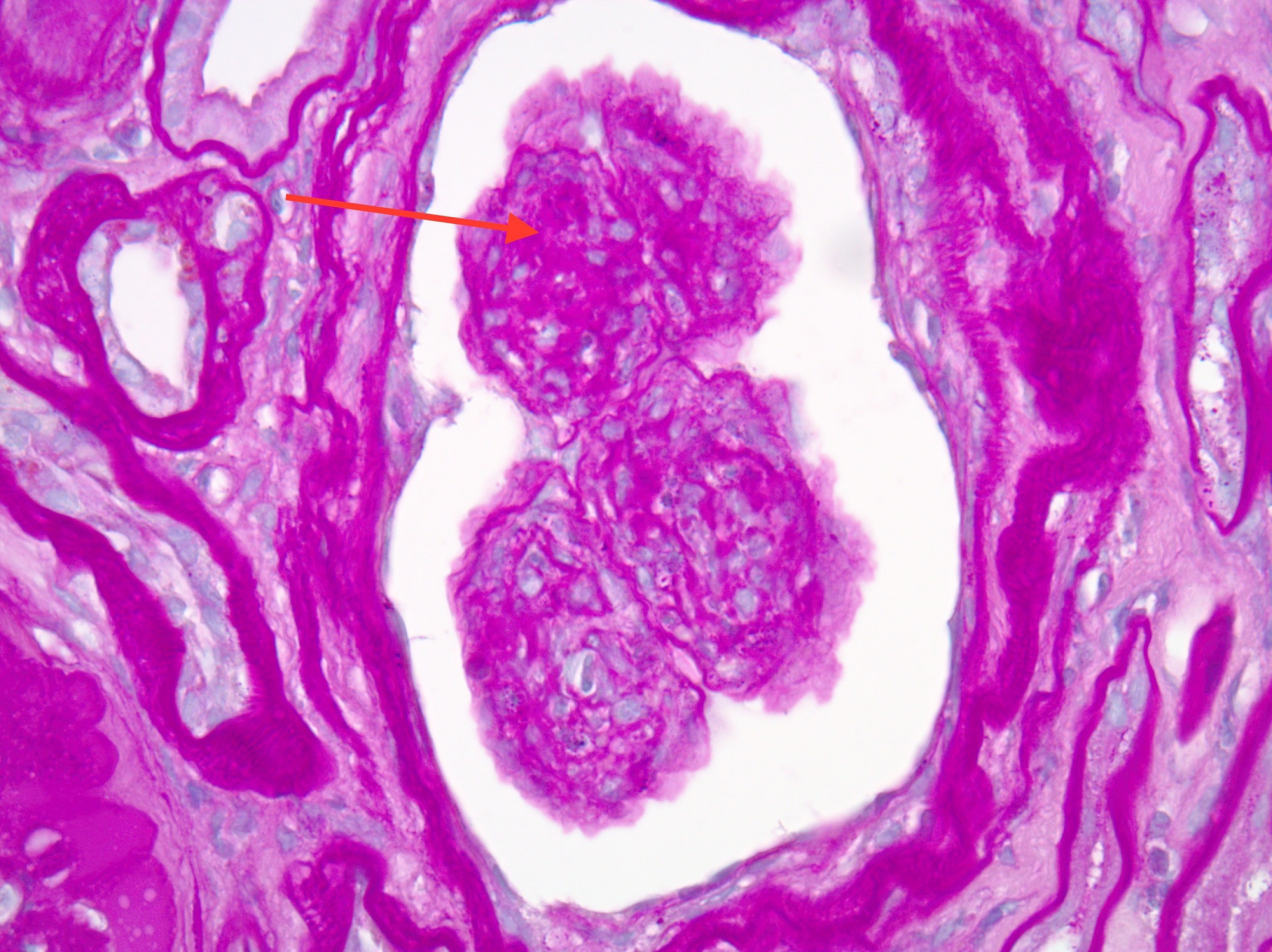
Light microscopy of renal biopsy demonstrating nodular sclerosis with prominent hyalinization (as indicated by the red arrow) present in glomerular capillary loops

**Figure 3:**
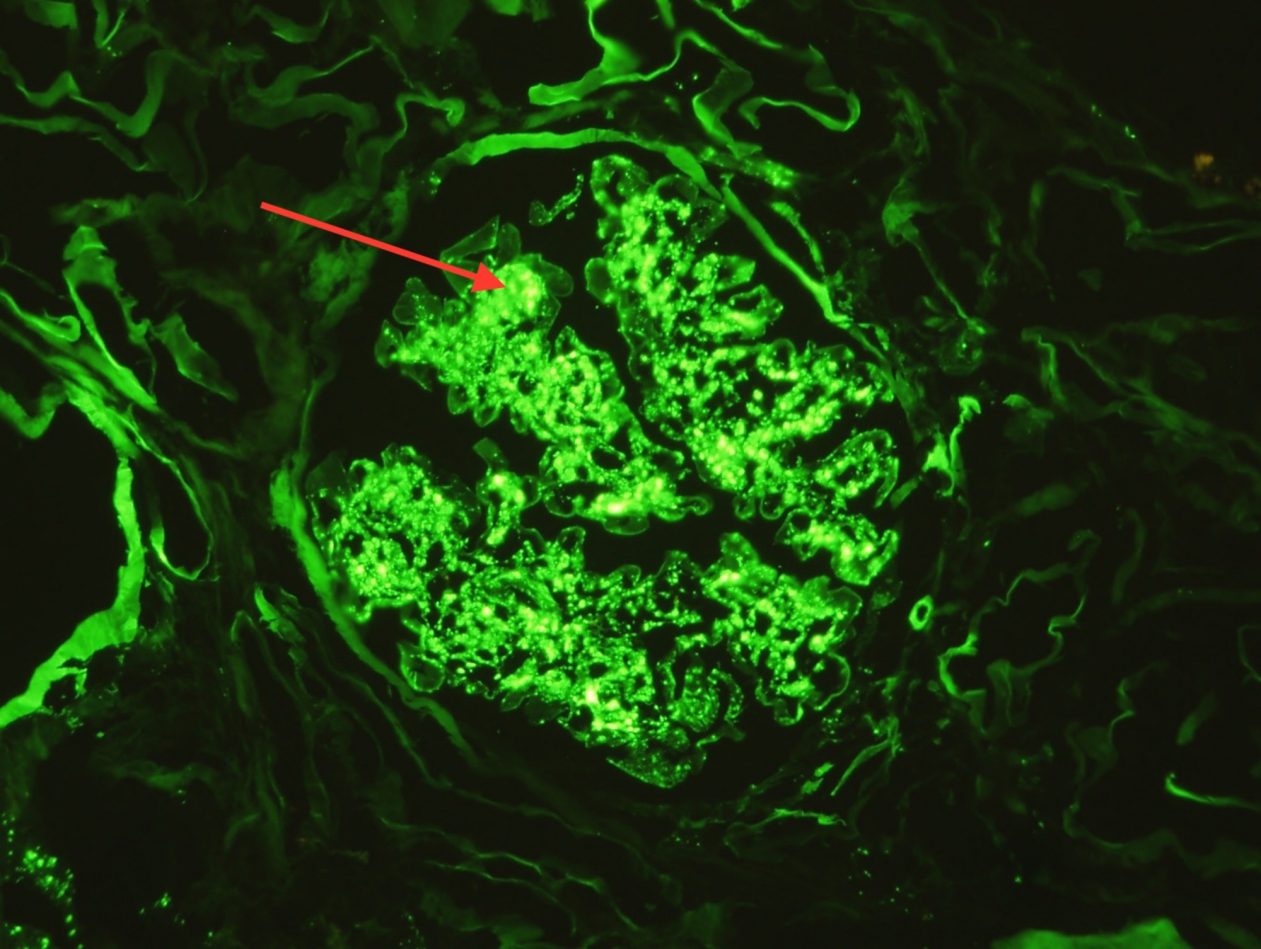
Immunofluorescence reveals significant complement C3 positive glomerular mesangial staining (as indicated by the red arrow)

Following a five-day clopidogrel washout, a renal biopsy was performed to further workup the etiology of this patient’s anuric AKI. Light microscopy of the kidney biopsy demonstrated diffuse endocapillary proliferative, exudative glomerulonephritis, and increase in mesangial cell counts, consistent with infection-related glomerulonephritis (Figure 1). In addition, moderate to severe nodular glomerulosclerosis with prominent hyalinization was present in glomerular capillary loops, consistent with chronic kidney disease secondary to diabetic nephropathy (Figure 2). Direct immunofluorescence staining for complement C3 revealed positive glomerular wall and coarsely granular mesangial staining (Figure 3). Per the biopsy report, other sites revealed tubular atrophy and interstitial fibrosis (moderate) and arterio/arteriolosclerosis (severe) (not shown). In short, the patient’s renal biopsy results were consistent with acute infection-related glomerulonephritis in the setting of CKD, likely secondary to HTN and diabetes.

Left ankle disarticulation (left guillotine below knee amputation (BKA)) was performed by vascular surgery to treat the patient’s left foot osteomyelitis 28 days after initial admission, after which he completed IV ertapenem for 72 hours at the recommendation of the infectious disease team. Despite an initial reported improvement in urine output to 200 cc daily (0.010 mL/kg/hr, normal range 1-2 mL/kg/hr) following the procedure, the patient remained hemodialysis-dependent on outpatient follow-up, reporting <100 cc urine output per day (0.05 mL/kg/hr, normal range 1-2 mL/kg/hr) per interview in the two weeks following surgery.

## Discussion

Infection-related glomerulonephritis (IRGN) secondary to osteomyelitis is rare, with few published cases [6-7]. When observed, it most often presents secondary to Staphylococcus aureus infection [8], with or without gram-negative organisms, as seen in this patient. Although we believe our patient’s IRGN to be related to Staphylococcus aureus infection, we cannot rule out Streptococcus-induced glomerulonephritis, as we were unable to attain anti-streptolysin O (ASO) titers. The most common sites for adult IRGN are the upper respiratory tract and skin [1]. While subclinical IRGN is four times more likely than symptomatic IRGN, symptomatic patients can present with symptoms of acute nephritic syndrome (hematuria, proteinuria, or peripheral edema) and renal dysfunction, with one study observing elevated creatinine (>4) in 67% of elderly patients aged >64 [9].

Definitive diagnosis is made via renal biopsy, where the most frequently observed histological pattern on light microscopy involves diffuse endocapillary proliferative with exudative glomerulonephritis [1]. Given the significant prevalence of age-associated co-morbidities, incidental findings on light microscopy are commonly observed and include underlying glomerulosclerosis, tubular atrophy, interstitial fibrosis, and arteriolar/arteriolosclerosis [1]. Immunofluorescence staining often reveals complement C3 deposition and granular mesangial and/or glomerular wall staining. Our renal biopsy results were consistent with these findings.

Due to its relative rarity, the precise mechanisms of IRGN have not been well-characterized, although current prevailing hypotheses characterize it as a type III hypersensitivity reaction, involving the formation and deposition of antigen-antibody immune complexes, which become trapped in glomerular capillaries or the mesangium, leading to complement activation and subsequent glomerular damage mediated by neutrophils [10]. Of note, although immunologically mediated, there has been no observed benefit in the use of steroids or other aggressive immunosuppressive therapies (azathioprine and cyclophosphamide) for IRGN management [8]. 

Instead, the recommended treatment of adult IRGN involves the aggressive management of the active infection with antibiotics and surgery if required [1]. Most patients with IRGN do not regain complete kidney function, and those with co-morbid diabetic glomerulosclerosis have particularly dismal outcomes; in one study of 11 patients, two had persistent renal dysfunction and nine went on to develop end-stage renal disease (ESRD) [4]. In our case, although IRGN generally has a poor outcome, we hypothesize prompt diagnosis and source control with surgical intervention may have increased the chances of renal recovery in this patient.

## Conclusions

In summary, physicians should be aware of IRGN as a rare but potentially severe complication of osteomyelitis. This case also underscores the value in prompt and aggressive management of active infections, including surgery if needed, in patients with renal dysfunction.
